# Supplementary material for: Exon sequencing mutation detection algorithm based on PCR matching
Source: PLoS One. 2020 Aug 13;15(8):e0236709. doi: 10.1371/journal.pone.0236709 (PMC7425927; doi:10.1371/journal.pone.0236709)
Supplement: Attachment — Submitted filename: PONE-D-19-33405_reviwer comments.docx [file pone.0236709.s001.docx]

**Reviewer Recommendation and Comments for Manuscript Number PONE-D-19-33405**

- An article in Nature Scientific reports (Ref given) has specified about primer-induced nucleotide labeling for massive sequencing on next-generation sequencing platforms by primer-induced sample labeling method for sequencing a large number of samples simultaneously on NGS. This article has specified on use of nucleotide labelling but the current article gives an insight into use of PCR primers for sequencing and with supporting algorithms. This is a novel idea and is applicable in this next gen sequencing era.
- The manuscript is well written and researched.
- The manuscript is acceptable to be published in PLoS One.

Ref: Guo, J., Cheng, T., Xu, H. *et al.* An efficient and cost-effective method for primer-induced nucleotide labeling for massive sequencing on next-generation sequencing platforms. *Sci Rep* **9,** 3125 (2019). https://doi.org/10.1038/s41598-019-38996-8
